# Diversity and composition of gut microbiota in healthy individuals and patients at different stages of hepatitis B virus-related liver disease

**DOI:** 10.1186/s13099-023-00549-w

**Published:** 2023-05-22

**Authors:** Meng-Ju Lin, Tung-Hung Su, Chieh-Chang Chen, Wei-Kai Wu, Shih-Jer Hsu, Tai-Chung Tseng, Sih-Han Liao, Chun-Ming Hong, Hung-Chih Yang, Chun-Jen Liu, Ming-Shiang Wu, Jia-Horng Kao

**Affiliations:** 1 School of Medicine, College of Medicine, National Taiwan University, Taipei, Taiwan; 2 Division of Gastroenterology and Hepatology, Department of Internal Medicine, National Taiwan University Hospital, 1 Chang-Te Street, Taipei, 10048 Taiwan; 3 Hepatitis Research Center, National Taiwan University Hospital, Taipei, Taiwan; 4 Department of Medical Research, National Taiwan University Hospital, Taipei, Taiwan; 5 Section of Gastroenterology, Department of Medicine, National Taiwan University Cancer Center, Taipei, Taiwan; 6 Division of Hospital Medicine, Department of Internal Medicine, National Taiwan University Hospital, Taipei, Taiwan; 7 Graduate Institute of Clinical Medicine, College of Medicine, National Taiwan University, 1 Chang-Te Street, Taipei, 10048 Taiwan

**Keywords:** Hepatitis B virus, Resolved hepatitis B virus infection, Liver cirrhosis, Hepatocellular carcinoma, Gut microbiota, 16S rRNA sequencing, Metagenomics

## Abstract

**Background:**

Hepatitis B virus (HBV) causes chronic hepatitis B (CHB), liver cirrhosis, and hepatocellular carcinoma. The evolution of human gut microbiota during the progression of HBV-related liver diseases remains unclear. Therefore, we prospectively enrolled patients with HBV-related liver diseases and healthy individuals. Through 16S ribosomal RNA amplicon sequencing, we characterized the gut microbiota of the participants and predicted the functions of microbial communities.

**Results:**

We analyzed the gut microbiota of 56 healthy controls and 106 patients with HBV-related liver disease [14 with resolved HBV infection, 58 with CHB, and 34 with advanced liver disease (15 with liver cirrhosis and 19 with hepatocellular carcinoma)]. Patients with HBV-related liver disease exhibited a higher degree of bacterial richness (all *P* < 0.05) than did healthy controls. Beta diversity analyses revealed a distinct clustering pattern between healthy controls and patients with HBV-related liver disease (all *P* < 0.05). The composition of bacteria (from the phylum level to the genus level) varied across the stages of liver disease. Linear discriminant analysis effect size revealed multiple taxa that differ significantly in abundance between healthy controls and patients with HBV-related liver disease; however, fewer differences were observed among patients with resolved HBV infection, those with CHB, and those with advanced liver disease. The ratio of Firmicutes to Bacteroidetes was increased in all three patient groups compared with the ratio in healthy controls (all *P* < 0.001). The analysis of the sequencing data by using PICRUSt2 revealed the changes in microbial functions with disease progression.

**Conclusions:**

The diversity and composition of gut microbiota appear to vary significantly between healthy controls and patients at different stages of HBV-related liver disease. The understanding of gut microbiota may provide novel therapeutic options in these patients.

**Supplementary Information:**

The online version contains supplementary material available at 10.1186/s13099-023-00549-w.

## Background

An estimated 296 million people are chronically infected with hepatitis B virus (HBV) worldwide, with 1.5 million new infections each year [1]. Thus, HBV infection remains a major threat to public health. Patients infected with HBV can develop acute or chronic hepatitis, liver cirrhosis (LC), and hepatocellular carcinoma (HCC) [1]. The term gut microbiota is referred to the microorganisms that colonize in the intestinal tract. The number of genes in the gut microbiota genome is 150-fold higher than in the human genome [2]. Accumulating evidence suggests a strong association between the liver and the intestines [3, 4]. The liver secretes primary bile acids and antimicrobial molecules into the intestinal lumen to facilitate digestion and control intestinal bacterial overgrowth. The portal system carries intestinal microbial products such as microbe-associated molecular patterns and endotoxins to the liver, facilitating the generation of a proinflammatory state [5].

Multiple studies have explored the alterations in the composition of microbiota in various liver diseases, including LC, alcoholic liver disease, and nonalcoholic fatty liver disease [6]. Gut microbiota may play a role in the pathogenesis of various HBV-related liver diseases [6]. Alterations have been demonstrated in the composition of gut microbiota in patients with HBV-related liver disease; these alterations are associated with a reduction in the abundance of beneficial bacteria and an increase in the growth rate of pathogenic species [7–11]. Nonetheless, the composition of intestinal microbiota may be affected by various host and environmental factors such as physical and mental health, medication, diet, and exposome [12]; therefore, the literature remains inconclusive.

A functional cure for chronic hepatitis B (CHB) is achieved through the seroclearance of hepatitis B surface antigen (HBsAg) [1]. The composition of microbiota in patients with resolved HBV infection (resolved HBV) remains unknown. Therefore, we compared the gut microbiota characteristics at different stages of HBV-related liver diseases (resolved HBV, CHB, and advanced stages of liver diseases) to identify potential taxonomic biomarkers and microbial functional profiles and thus improve our understanding of the gut microbiota in these patients.

## Results

### Clinical characteristics of the study population

In this study, we analyzed the gut microbiota of 56 healthy controls and 106 patients with HBV-related liver diseases. Among these patients, 14 had resolved HBV, 58 had CHB, and 34 had advanced liver disease (15 had LC, whereas 19 had HCC). Of the patients with HCC, 12 (63%) had a history of LC. The baseline characteristics of the participants are summarized in Table 1. The median age was 28 years for healthy controls, 57 years for resolved HBV patients, 51 years for CHB patients, and 62 years for advanced liver disease patients (*P* < 0.0001); the corresponding proportions of men were 32%, 50%, 66%, and 82% (*P* < 0.0001), respectively. In general, the levels of alanine aminotransferase (ALT), aspartate aminotransferase (AST), blood urea nitrogen, creatinine, and fasting blood glucose were higher in patients with resolved HBV, those with CHB, and those with advanced liver disease than in healthy controls (all *P* < 0.0001). Patients with advanced liver disease had lower platelet counts than did those with resolved HBV or CHB (*P* = 0.0019).

**Table 1:** Demographics and clinical characteristics of the study population

Parameters	Healthy control (n = 56)	Resolved HBV (n = 14)	Chronic hepatitis B (n = 58)	Advanced liver disease (n = 34)	*P* value
Age (years)	28 (24–36)	57 (53–65)	51 (42–59)	62 (56–67)	< 0.0001^abcf^
Sex (male)	18 (32)	7 (50)	38 (66)	28 (82)	< 0.0001
BMI (kg/m^2^)		23.3 (22.1–25.8)	23.5 (20.6–27.0)	24.2 (20.8–26.4)	0.9131
HBsAg positive		0 (0)	58 (100)	33 (97.1)	< 0.0001
HBeAg positive		0 (0)	19 (32.8)	5 (14.7)	0.0153
ALT (U/L)	6 (5–10)	12 (10–20)	29.5 (18–76)	25.5 (18–42)	< 0.0001^abcde^
AST (U/L)	12.5 (9–15)	18.5 (17–25)	27 (19–57)	28 (18–54)	< 0.0001^abc^
BUN (mg/dL)	10.3 (8.9–12.5)	14.4 (10.3–15.7)	13.5 (10.1–15.9)	16.3 (13.1–18.8)	< 0.0001^bcf^
CRE (mg/dL)	0.6 (0.5–0.7)	0.7 (0.7–0.8)	0.8 (0.7–0.9)	0.9 (0.8–1)	< 0.0001^abc^
GLU-AC (mg/dL)	73.5 (67–80)	98 (89–115)	91 (86–96)	103 (90–141)	< 0.0001^abc^
Platelet count (10^9^/L)		230 (211–245)	215 (170–249)	180 (113–207)	0.0019^ef^
T-CHO (mg/dL)		180 (159–225)	191 (146–215)	178 (142–199)	0.5517
HDL (mg/dL)		42 (38–62)	51 (42–56)	47 (39–52)	0.8869
LDL (mg/dL)		105 (89–124)	118 (83–142)	109 (98–132)	0.7194
TG (mg/dL)		145 (113–193)	84 (74–133)	103 (78–127)	0.1166

Data are expressed as median (interquartile range) or number (percentage)

BMI, body mass index; HBsAg, hepatitis B surface antigen; HBeAg, hepatitis B e antigen; ALT, alanine aminotransferase; AST, aspartate aminotransferase; BUN, blood urea nitrogen; CRE, creatinine; GLU-AC, fasting blood glucose; T-CHO, total cholesterol; HDL, high-density lipoprotein; LDL, low-density lipoprotein; TG, triglyceride

a, significant between healthy control and patients with resolved HBV

b, significant between healthy control and patients with chronic hepatitis B

c, significant between healthy control and patients with advanced liver disease

d, significant between patients with resolved HBV and those with chronic hepatitis B

e, significant between patients with resolved HBV and those with advanced liver disease

f, significant between patients with chronic hepatitis B and those with advanced liver disease

### Overview of the sequencing data

The 16S ribosomal RNA (rRNA) gene V3–V4 amplicon sequencing yielded 15,801,571 reads, and the average number of sequence reads per sample was 97,540 (minimum: 46,986; maximum: 183,523). To account for sample size–related variations, rarefaction was performed to normalize the reads to 46,986 reads per sample. A total of 7,504 amplicon sequence variants (ASVs) were retained after rarefaction.

### Differences in gut microbiome structures

**Fig. 1:**
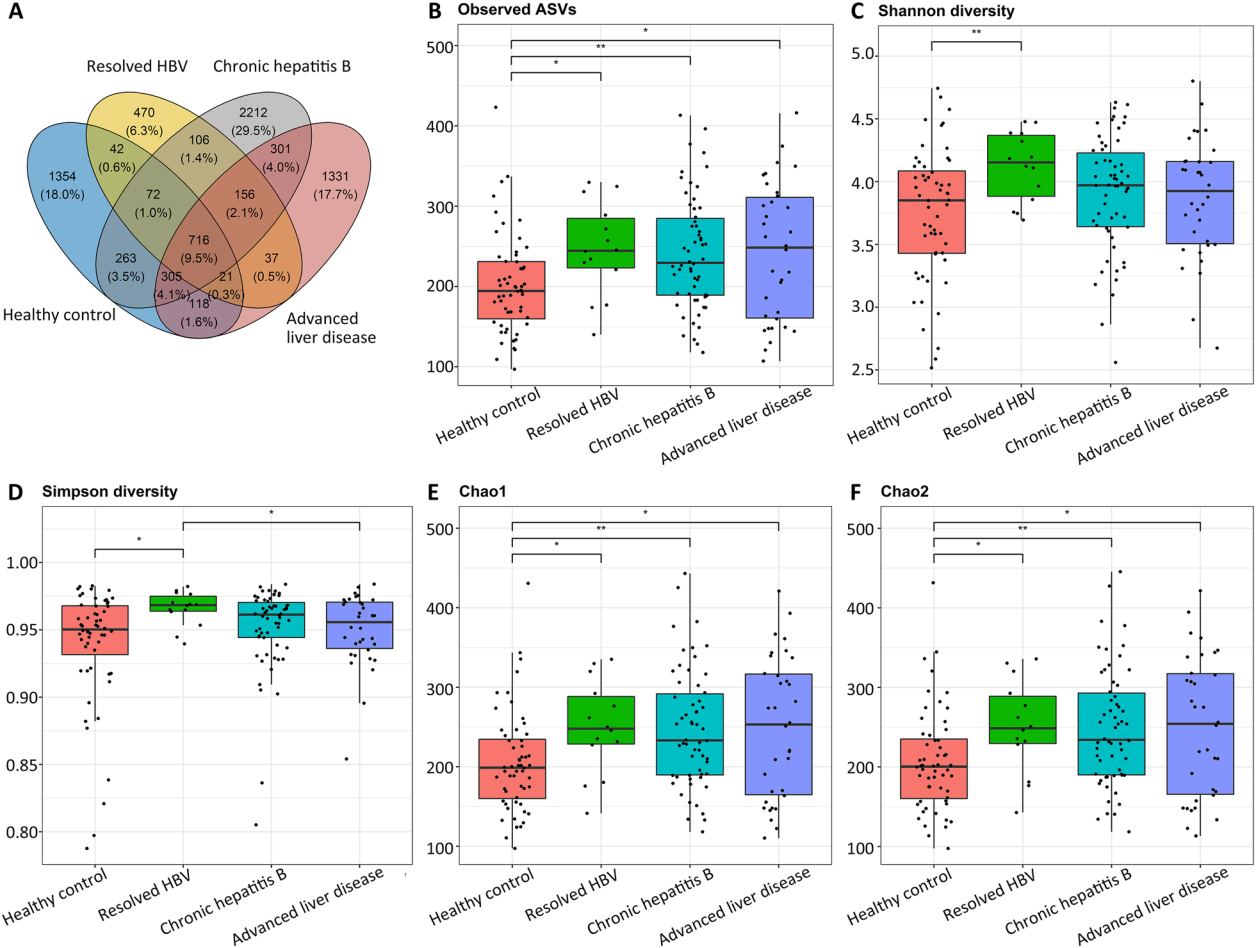
Comparisons of bacterial diversity and richness between healthy controls and patients with resolved HBV, CHB, and advanced liver disease. **A** A Venn diagram displays the unique and shared ASVs among the four groups. **B** The healthy controls harbored the lowest observed ASVs. Alpha diversity indices including **C** Shannon diversity, **D** Simpson diversity, **E** Chao1 index, and **F** Chao2 index revealed the differences in richness and evenness between groups. * means *P* < 0.05 and ** means *P* < 0.01

The common and unique ASVs among the different stages of liver diseases and the number of observed ASVs are presented in Fig. 1A, B. Alpha diversity matrices were calculated on the basis of rarefied ASVs. The estimated values of Simpson and Shannon diversities revealed higher degrees of bacterial richness and evenness in patients with resolved HBV than in healthy controls (Fig. 1C, D). Chao1 and Chao2 indices indicated a higher degree of species richness in patients with HBV-related liver disease than in healthy controls (all *P* < 0.05; Fig. 1E, F).

**Fig. 2:**
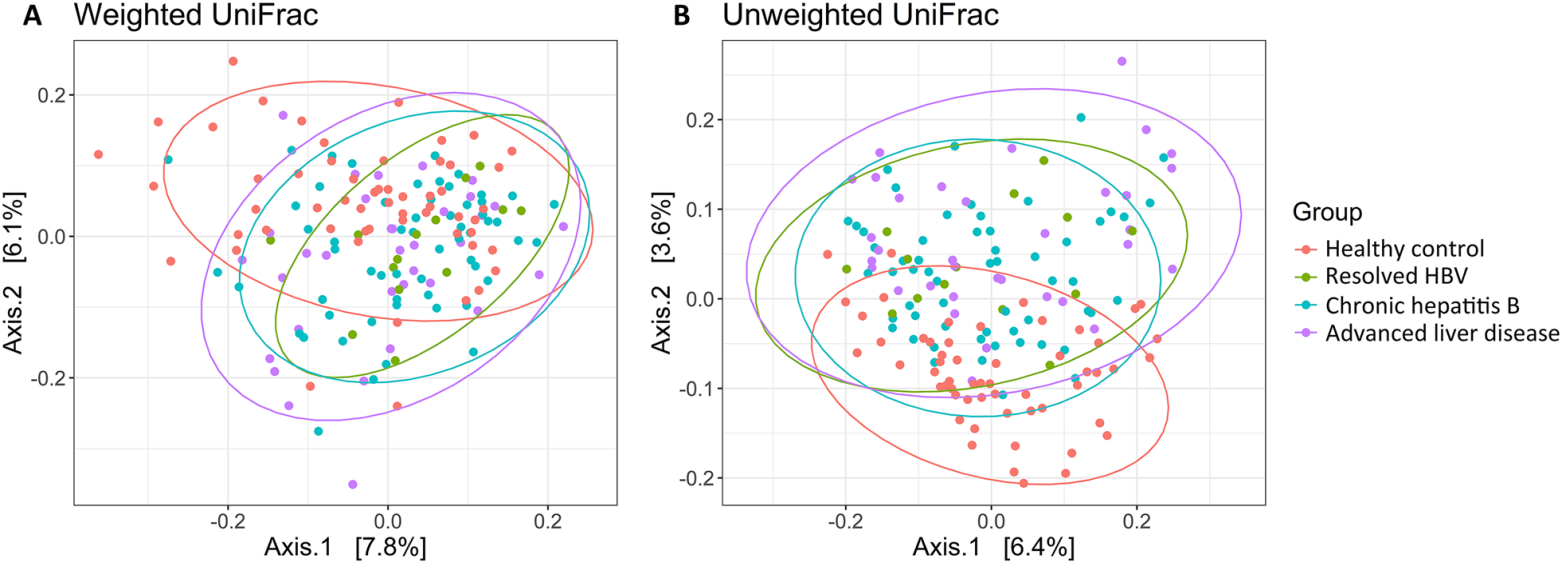
Beta diversity indices of the fecal microbiota. **A** PCoA plot of bacterial beta diversity based on the weighted UniFrac distance **B** PCoA plot of bacterial beta diversity based on the unweighted UniFrac distance. Beta diversity analyses revealed a distinct clustering pattern between healthy controls and patients with HBV-related liver disease

Principal coordinate analysis (PCoA) performed using weighted and unweighted UniFrac distances revealed distinct clusters between healthy controls and the aforementioned three patient groups (Fig. 2A, B). The adonis2 function, which was used to evaluate between-group dissimilarities and adjust for the possible confounding effect of age in the multivariate model, revealed a nonsignificant effect of age (*P* = 0.671 for weighted UniFrac distance and *P* = 0.146 for unweighted UniFrac distance) on microbiota composition compared with the significant effect of disease status (*P* = 0.001 for both distances). Pairwise comparisons performed using the permutational multivariate analysis of variance (PERMANOVA) indicated significant differences in bacterial composition between patients with resolved HBV and healthy controls, between patients with CHB and healthy controls, and between patients with advanced liver disease and healthy controls (all q < 0.05 for weighted and unweighted UniFrac distances; Additional file 1). The composition of fecal microbiota communities was relatively similar among the three patient groups.

### Bacterial abundance and taxonomic distribution

**Fig. 3:**
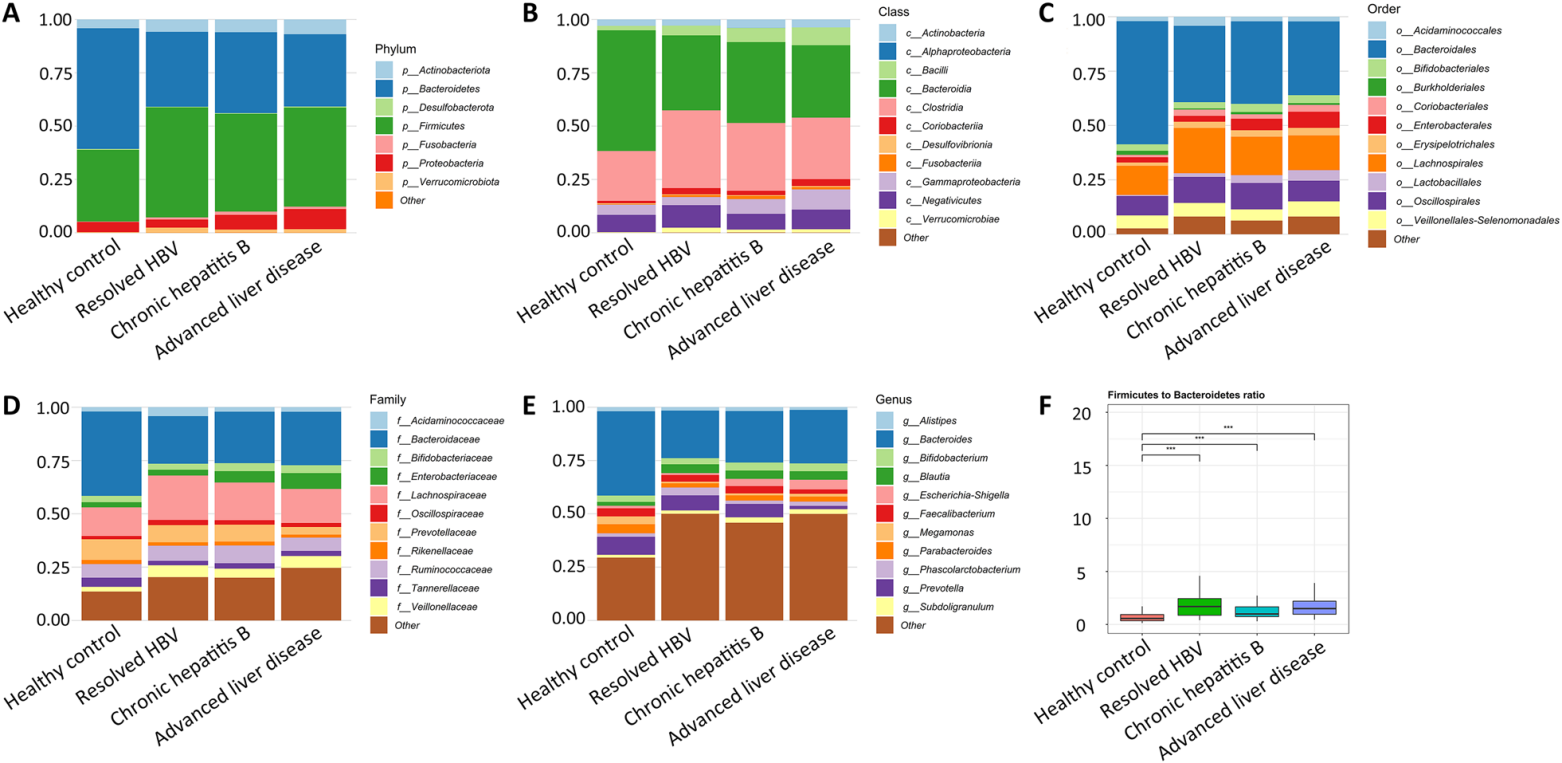
Distribution of the predominant bacterial taxa at different taxonomic levels. **A**, **B**, **C**, **D**, **E** represent phylum, class, order, family, and genus levels, respectively. **F** The ratio of Firmicutes to Bacteroidetes (F/B ratio) was increased in patients with resolved HBV, CHB, and advanced liver disease compared to healthy controls. *** means *P* < 0.001

The most abundant phyla in the study cohort were Bacteroidetes (44.7%), Firmicutes (41.4%), Proteobacteria (6.0%), Actinobacteriota (5.5%), and Verrucomicrobiota (1.0%). The most abundant genera were *Bacteroides* (30.3%), *Prevotella* (6.5%), *Bifidobacterium* (3.4%), *Faecalibacterium* (3.3%), *Blautia* (3.1%), *Parabacteroides* (3.1%), *Escherichia-Shigella* (2.6%), and *Megamonas* (2.0%). The taxonomic distribution of the predominant bacteria among the study groups is shown in Fig. 3A–E. The ratio of Firmicutes to Bacteroidetes (F/B ratio) was higher in all three patient groups than in healthy controls (all *P* < 0.001; Fig. 3F).

### Differences in gut microbiota compositions

**Fig. 4:**
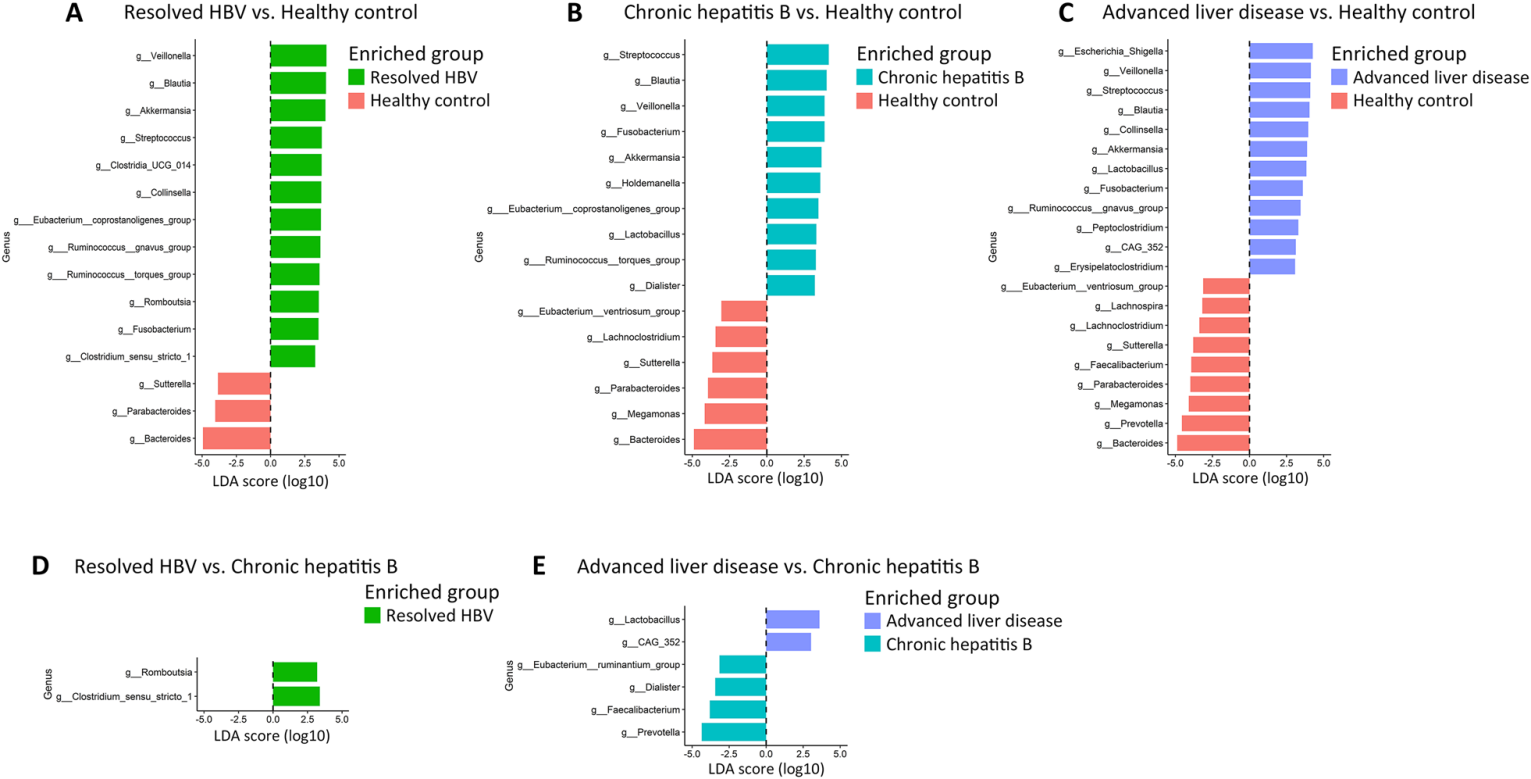
Differentially abundant genera between groups identified by LEfSe (only logarithmic LDA scores > 3.0 are shown). **A** Resolved HBV vs. Healthy controls. **B** Chronic hepatitis B vs. Healthy controls. **C** Advanced liver disease vs. Healthy controls. **D** Resolved HBV vs. Chronic hepatitis B. **E** Advanced liver disease vs. Chronic hepatitis B

Linear discriminant analysis (LDA) effect size (LEfSe) was used to identify specific taxonomic biomarkers for the study groups. The cutoff value of the logarithmic linear discriminant analysis (LDA) score was set at 3.0 to determine the major taxonomic differences between the following groups: (1) healthy controls and patients with resolved HBV, (2) healthy controls and patients with CHB, (3) healthy controls and patients with advanced liver disease, (4) patients with CHB and those with resolved HBV, and (5) patients with CHB and those with advanced liver disease. Significant differences were observed between healthy controls and patients with HBV-related liver disease (Fig. 4A–E).

Regarding phylum-level comparisons, Firmicutes, Verrucomicrobiota, and Fusobacteria were enriched and Bacteroidetes was depleted in the three patient groups compared with the findings in healthy controls. Furthermore, Proteobacteria and Actinobacteriota were enriched in patients with advanced liver disease. The abundance of Proteobacteria was higher in patients with advanced liver disease than in those with CHB (Additional file 2).

Genus-level comparisons between patients with resolved HBV and healthy controls (Fig. 4A) revealed that the most enriched genera in patients with resolved HBV were *Veillonella* (LDA score = 4.08; *P* < 0.001), *Blautia* (LDA score = 4.05; *P* = 0.003), *Akkermansia* (LDA score = 4.01; *P* = 0.004), *Streptococcus* (LDA score = 3.74; *P* < 0.001), and *Clostridia* UCG-014 (LDA score = 3.73; *P* = 0.017), whereas the most enriched genera in healthy controls were *Bacteroides* (LDA score = 4.94; *P* = 0.004), *Parabacteroides* (LDA score = 4.04; *P* = 0.008), and *Sutterella* (LDA score = 3.84; *P* < 0.001).

Genus-level comparisons between patients with CHB and healthy controls (Fig. 4B) revealed that the most enriched genera in patients with CHB were *Streptococcus* (LDA score = 4.15; *P* < 0.001), *Blautia* (LDA score = 4.01; *P* = 0.001), *Veillonella* (LDA score = 3.87; *P* < 0.001), *Fusobacterium* (LDA score = 3.87; *P* = 0.007), and *Akkermansia* (LDA score = 3.66; *P* = 0.012), whereas the most enriched genera in healthy controls were *Bacteroides* (LDA score = 4.89; *P* < 0.001), *Megamonas* (LDA score = 4.17; *P* = 0.005), *Parabacteroides* (LDA score = 3.95; *P* < 0.001), *Sutterella* (LDA score = 3.65; *P* = 0.001), and *Lachnoclostridium* (LDA score = 3.44; *P* = 0.005).

Genus-level comparisons between patients with advanced liver disease and healthy controls (Fig. 4C) revealed that the most enriched genera in patients with advanced liver disease were *Escherichia-Shigella* (LDA score = 4.26; *P* = 0.018), *Veillonella* (LDA score = 4.13; *P* < 0.001), *Streptococcus* (LDA score = 4.10; *P* < 0.001), *Blautia* (LDA score = 4.04; *P* < 0.001), and *Collinsella* (LDA score = 3.96; *P* = 0.002), whereas the most enriched genera in healthy controls were *Bacteroides* (LDA score = 4.87; *P* = 0.001), *Prevotella* (LDA score = 4.56; *P* < 0.001), *Megamonas* (LDA score = 4.09; *P* = 0.003), *Parabacteroides* (LDA score = 3.98; *P* < 0.001), and *Faecalibacterium* (LDA score = 3.92; *P* = 0.002).

Genus-level comparisons between patients with resolved HBV and those with CHB (Fig. 4D) revealed that *Clostridium* sensu stricto 1 (LDA score = 3.39; *P* = 0.012) and *Romboutsia* (LDA score = 3.20;* P* = 0.041) were enriched in patients with resolved HBV, whereas no taxon was enriched in patients with CHB.

Finally, genus-level comparisons between patients with CHB and those with advanced liver disease (Fig. 4E) revealed that *Prevotella* (LDA score = 4.36; *P* = 0.013), *Faecalibacterium* (LDA score = 3.81; *P* = 0.026), *Dialister* (LDA score = 3.44; *P* = 0.003), and *Eubacterium* ruminantium group (LDA score = 3.15;* P* = 0.039) were enriched in patients with CHB, whereas *Lactobacillus* (LDA score = 3.60; *P* = 0.028) and CAG-352 (LDA score = 3.03; *P* = 0.027) were enriched in patients with advanced liver disease.

**Fig. 5:**
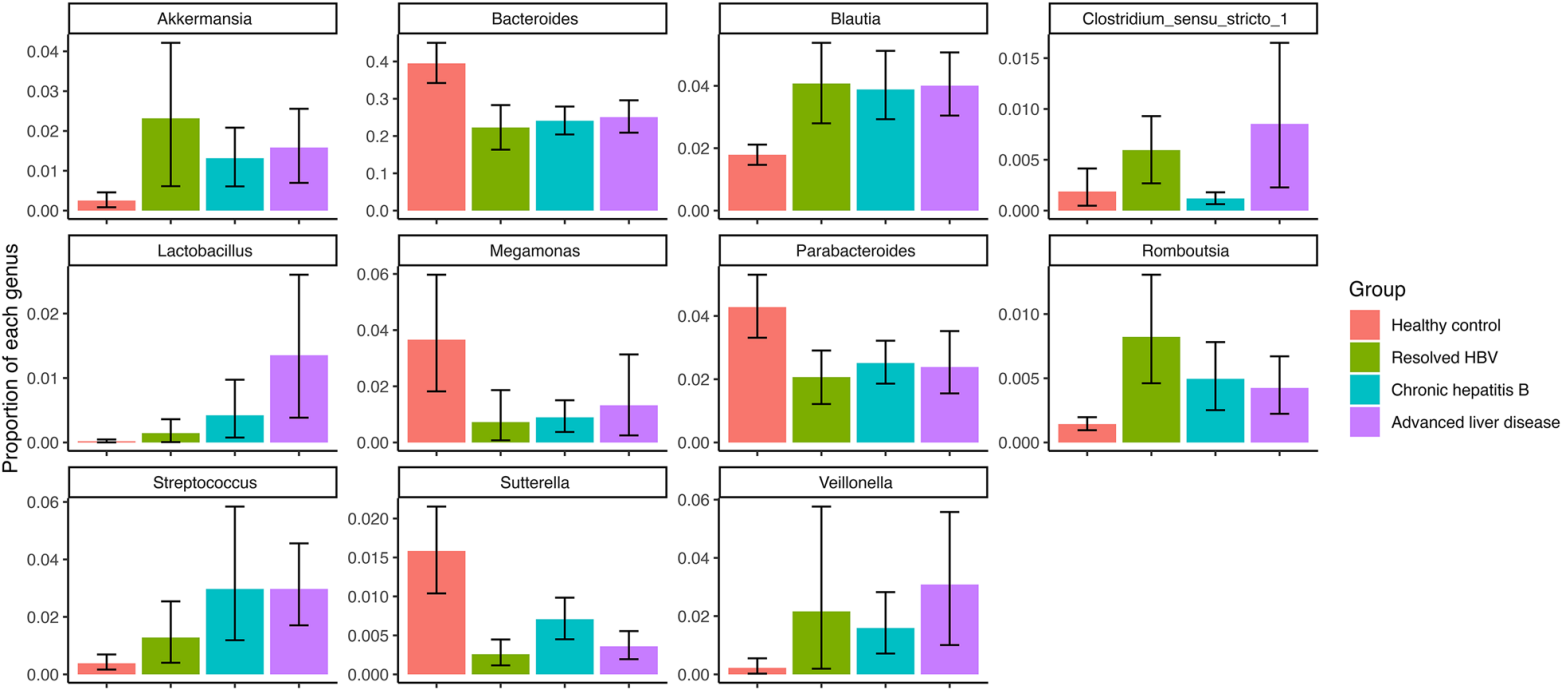
Mean relative abundance of selected genera

Fig. 5 shows the mean relative abundance of the following 11 selected genera: *Bacteroides*, *Megamonas*, *Parabacteroides*, and *Sutterella*, which were enriched in healthy controls; *Akkermansia*, *Blautia*, *Lactobacillus*, *Streptococcus*, and *Veillonella*, which were enriched in the patient groups (all *P* for trend < 0.01); and *Clostridium* sensu stricto 1 and *Romboutsia*, which had higher abundances in patients with resolved HBV than in those with CHB.

### Functional analysis based on 16S rRNA sequencing data

**Fig. 6:**
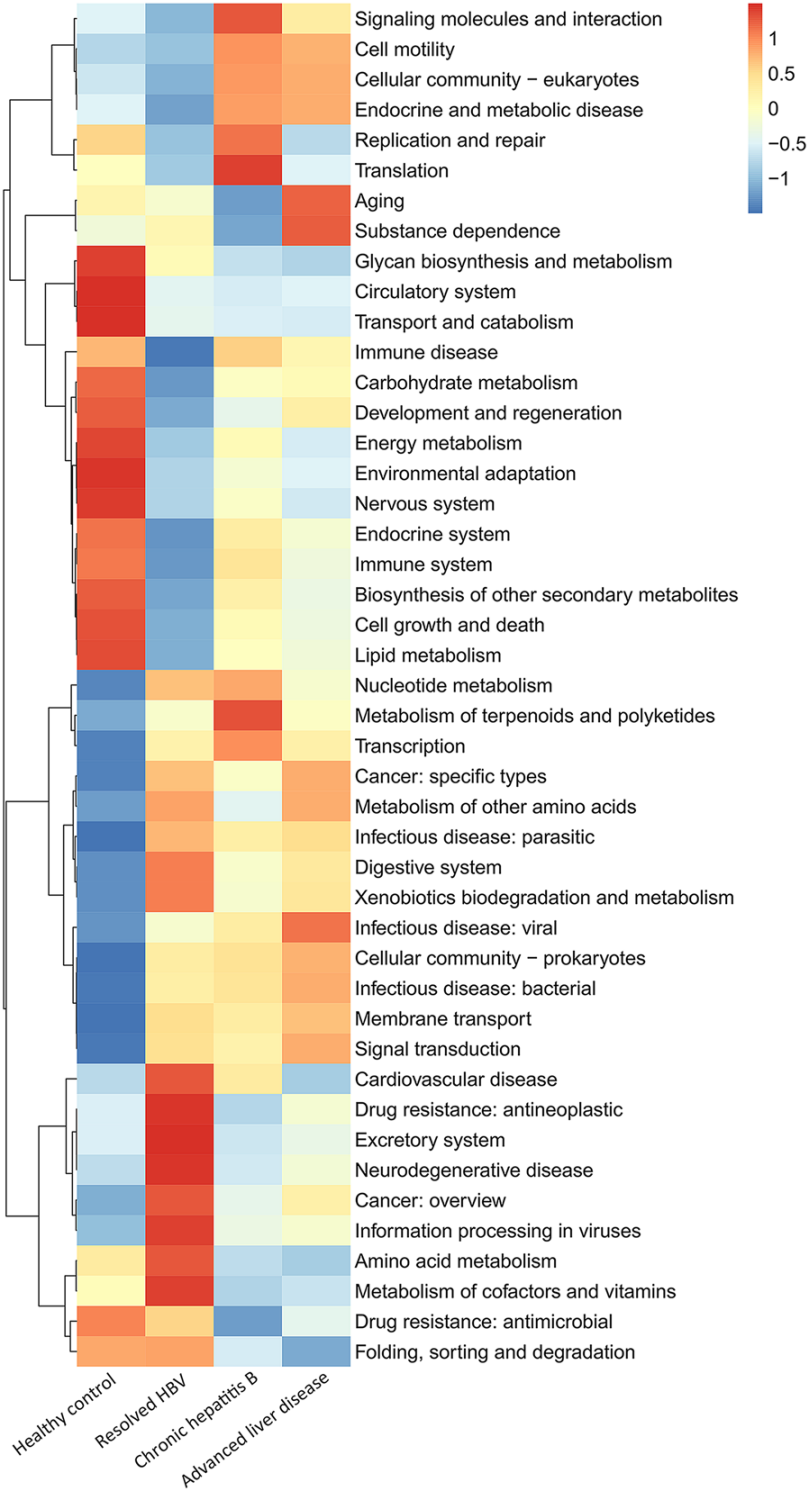
Differences of predicted microbial functions with PICRUSt2 analysis based on 16S rRNA sequencing. The microbial functions were clustered based on the similarities of expression pattern

We identified 45 metabolic functions that were classified as Level 2 of the Kyoto Encyclopedia of Genes and Genomes (KEGG) Orthologs (Additional file 3). A metagenome function prediction analysis performed using the Phylogenetic Investigation of Communities by Reconstruction of Unobserved States 2 (PICRUSt2) tool revealed alterations in bacterial functions with disease progression. A total of 28 differentially expressed pathways were identified (Additional file 4). A heatmap was generated to visualize the differential expression levels of Level 2 KEGG pathways (Fig. 6). The pathways enriched with disease progression were involved in membrane transport, cancer, bacterial infection, viral infection, transcription, endocrine and metabolic disease, and cell motility. By contrast, pathways enriched in healthy controls were involved in cell growth and death, endocrine system, carbohydrate, glycan, lipid, terpenoids, and polyketides metabolism, transport and catabolism, energy metabolism, circulatory system, nervous system, and immune disease. Notably, pathways involved in the metabolism of cofactors and vitamins and the development of antineoplastic drug resistance exhibited the highest levels of enrichment in patients with resolved HBV.

### Subgroup analysis in patients with CHB

Patients with CHB were further divided into the following four groups (representing the four phases of HBV natural history) according to the presence of the hepatitis B e antigen (HBeAg) and the level of ALT: HBeAg (+) chronic HBV infection, HBeAg (+) chronic hepatitis B, HBeAg (−) chronic HBV infection, and HBeAg (−) chronic hepatitis B. The alpha and beta diversities were compared between the four groups and patients with resolved HBV. The values of the alpha diversity indices, such as Shannon diversity, Simpson diversity, Chao1, and Chao2, and the number of observed ASVs were lower in patients with HBeAg (−) chronic hepatitis B (Additional file 5). PCoA plots generated on the basis of weighted and unweighted UniFrac distances revealed no distinct separation between the aforementioned four groups and patients with resolved HBV (all *P* > 0.05; Additional file 6 and Additional file 7).

## Discussion

HBV infection remains difficult to cure despite the development of antiviral therapies [13]. Therefore, targeting gut microbiota has emerged as a novel therapy for HBV infection and its complications [14]. In this study, we identified the alterations in the diversity and composition of gut microbiota between healthy controls and patients with resolved HBV, CHB, or advanced liver disease to elucidate the relationship between the composition of gut microbiota and the progression of HBV-related liver disease.

In this study, healthy controls were younger than patients with HBV-related liver disease. A large cohort-based study revealed no association between microbial alpha diversity and age in a cohort from China, in contrast to the findings in cohorts from the United States, the United Kingdom, and Colombia [15]. Our findings also indicated minimal confounding effect of age on the microbiota composition. Thus, the differences in microbiota composition between healthy controls and the three patient groups might have been due to the different disease statuses, rather than the baseline characteristics of the patients.

In our study, the species richness estimated using Chao1 and Chao2 indices and the observed ASVs were increased in the three patient groups compared with those in healthy controls. Shannon and Simpson diversities were similar between the healthy controls, patients with CHB, and patients with advanced liver disease but higher in patients with resolved HBV than in healthy controls. By contrast, a study reported lower Chao1 index values and smaller operational taxonomic unit (OTU) numbers in patients with LC than in healthy controls and asymptomatic HBV carriers [8]. Furthermore, another study revealed lower Shannon diversity, Simpson diversity, and Chao1 index values in patients with LC than in those with early-stage HBV-related disease [7]. However, other studies have reported no significant between-group differences in alpha diversity [9, 16]. These discrepancies might be attributed to the heterogeneity in patient population.

The gut microbiome structure, assessed in terms of beta diversity, varied qualitatively and quantitatively between healthy controls and patients with HBV-related liver disease. Our finding is consistent with that reported by Zeng et al.[9] Beta diversity analyses performed using Bray − Curtis-based nonmetric multidimensional scaling also revealed alterations in microbiota composition in patients with HBV infection, those with CHB, and those with LC in another study [7].

The phyla consistently enriched in the three patient groups were Firmicutes, Verrucomicrobiota, and Fusobacteria; by contrast, Bacteroidetes was depleted in the patient groups. Proteobacteria and Actinobacteriota were enriched in patients with advanced liver disease. Firmicutes and Bacteroidetes are two predominant phyla found in the gut environment. Bacteroidetes are gram-negative bacteria; the members of this phylum perform metabolic functions such as the degradation of proteins and complex sugar polymers. By contrast, Firmicutes are gram-positive bacteria. This phylum includes probiotic species such as *Lactobacillus* sp. and opportunistic pathogens such as *Clostridium difficile*.[17] A marked reduction in the abundance of Bacteroidetes was noted in patients with HBV-related LC [18, 19]. This reduction might have been because of the overgrowth of the pathogenic strains of Proteobacteria and Fusobacteria [19]. An increase in the Firmicutes to Bacteroidetes ratio, which is a marker of dysbiosis, was indicated to be associated with HBV-related LC [20]. In our study, the F/B ratio observed in patients with resolved HBV was more similar to the ratios observed in patients with CHB and those with advanced liver disease than to the ratio observed in healthy controls. This finding suggests the presence of dysbiosis in the patient groups.

The genera *Akkermansia*, *Veillonella*, *Blautia*, and *Streptococcus* were consistently enriched, whereas the genera *Sutterella*, *Parabacteroides*, and *Bacteroides* were depleted in patients with HBV-related liver disease. *Bacteroides* participates in carbohydrate and lipid metabolism and contributes to short-chain fatty acid (SCFA) synthesis [21]. In addition, *Bacteroides* plays a role in the intestinal immune network by activating intestinal dendritic cells, which help control the overgrowth of pathogenic strains and maintain the integrity of the intestinal epithelial barrier [22, 23]. Changes in the microenvironment of the intestinal tract with the progression of liver diseases may reduce the abundance of *Bacteroides* and facilitate the colonization of opportunistic pathogens. *Veillonella* has been reported to be enriched in patients with advanced-stage HBV infection [7–10, 24]. This bacterial genus is associated with inflammation and can thus serve as a pathogenic biomarker of chronic liver inflammation [25]. The abundance of another opportunistic pathogen, *Streptococcus*, was reported to be higher in patients with LC than in healthy controls [8, 10].

*Akkermansia* was reported to be most enriched in patients with LC, with relatively low abundance in the healthy controls, patients with CHB, and patients with HCC [9]. However, we found that *Akkermansia* was generally enriched in patients with HBV-related liver disease. A key species of *Akkermansia* is *A. muciniphila*, which is a mucin-degrading bacterium that is associated with a lower risk of obesity. This species also performs several metabolic functions and maintains gastrointestinal mucosal integrity [26, 27]. Future studies should aim to identify the factors contributing to the increased abundance of *Akkermansia* in patients with HBV-related CHB, LC, or HCC and the role of this species in the progression of the aforementioned disease.

*Prevotella*, *Faecalibacterium*, *Dialister*, and *Eubacterium* ruminantium group were enriched in patients with CHB, whereas *Lactobacillus* and CAG-352 were enriched in those with advanced liver disease. *Lactobacillus* spp. are commonly used probiotics and are effective in ameliorating gastrointestinal problems such as gastrointestinal tract infections and diarrhea, inflammatory bowel disease, nonalcoholic fatty liver disease, and nonalcoholic steatohepatitis [28]. However, consistent with our findings, studies have reported an association between the increased abundance of *Lactobacillus* and the progression of HBV-related liver diseases, such as decompensated LC and HCC [8, 16, 24]. Further studies on the metabolic functions of *Lactobacillus* in HBV-related liver disease are needed.

In this study, the abundances of *Clostridium* sensu stricto 1 and *Romboutsia* were higher in patients with resolved HBV than in those with CHB. Lu et al. demonstrated an increase of the abundance of *Clostridium* sensu stricto 1 in patients with CHB receiving entecavir for 8 weeks [29]. Furthermore, an animal study revealed that the abundance of *Clostridium* sensu stricto was negatively correlated with the levels of HBsAg and HBeAg after entecavir treatment [30]. The family *Clostridiaceae* includes bacteria that produce SCFAs, such as acetate, butyrate, and propionate, through carbohydrate fermentation [31]. SCFAs participate in multiple metabolic functions and function as anti-inflammatory agents [32]. The genus *Romboutsia* has recently been separated from the genus *Clostridium* and has been described as a nonpathogenic commensal found in the gastrointestinal tract [33]. Thus, the increased abundances of *Clostridium* and *Romboutsia* species may indicate reduced liver inflammation and HBsAg seroclearance.

Notably, although patients with resolved HBV achieved the seroclearance of HBsAg and normalization of hepatic inflammation either spontaneously or through antiviral therapies, the alterations in their gut microbiota persisted and were more similar to the microbial profiles noted in patients with chronic liver diseases than to the profiles noted in healthy controls. This might be because the long-term chronic infection had already shaped the composition of patients’ gut microbiota and the alterations in gut microbiota composition after HBsAg seroclearance might not have been sufficiently significant to reach the preset LDA cutoff score of 3.0.

PICRUSt2 analysis revealed that the metabolic function of viral infectious disease was increasingly activated with disease severity in a dose-dependent manner. Unlike two relevant studies [8, 9], our study indicated that the lipid metabolism pathway was enriched in healthy controls, possibly because of the higher abundance of Bacteroidetes in these individuals than in the patient groups. Furthermore, we identified 28 differentially expressed metabolic pathways; most of these pathways varied between healthy controls and the three patient groups. Therefore, the interactions between microbiota composition and metagenomic functional profiles might have contributed to the progression of HBV-related disease.

Our study has some limitations. First, because of the cross-sectional design of this study, we could obtain limited data regarding the role of microbiota in the progression of HBV-related liver diseases. Second, the smaller sample size of patients with resolved HBV allowed us to draw only preliminary conclusions. Finally, the results obtained through the random sampling of fecal samples and the subsequent rarefaction of reads might not have represented the actual microbiota composition in the gastrointestinal tract. To offer in-depth species-level information and clarify metabolic functions, shotgun metagenomic sequencing should be performed in future studies aimed at elucidating the mechanisms underlying the pathogenesis of HBV-related liver diseases.

## Conclusions

The diversity and composition of gut microbiota appear to vary significantly between healthy individuals and patients at different stages of HBV-related liver disease. Gut dysbiosis persists even after the seroclearance of HBsAg (resolved HBV). In the future, studies should be conducted on the shift and evolution of microbiota in CHB, LC, HCC, and resolved HBV patients to elucidate the mechanisms underlying the pathogenesis of HBV-related liver disease and develop effective microbiome-based treatment strategies for these diseases.

## Methods

### Patients

Patients with HBV-related liver disease (resolved HBV, CHB, or advanced liver disease [LC or HCC]) were prospectively enrolled at National Taiwan University Hospital between 2019 and 2021. Healthy individuals with no known history of liver or gastrointestinal diseases, such as inflammatory bowel disorders, irritable bowel syndrome, or colitis, were enrolled in 2016.

Chronic HBV infection was confirmed on the basis of the presence of HBsAg for at least 6 months. Resolved HBV was defined as seronegativity for HBsAg but seropositivity for hepatitis B core antibody (anti-HBc) and normal ALT levels in patients with a known history of CHB. LC was diagnosed on the basis of histological findings, a fibroscan result of ≥ 12 kPa, or the observation of a nodular liver surface, a coarse liver parenchymal texture, and narrowed vessels with irregular intrahepatic vessel contours on ultrasonography [34]. HCC was diagnosed on the basis of pathological findings or one typical dynamic imaging study according to the American Association for the Study of Liver Diseases guideline [35]. Patients were excluded if (1) they had a history of hepatitis C, hepatitis D, or human immunodeficiency virus coinfection; (2) they had autoimmune disorders or used any immunomodulatory drug; (3) they had a history of antibiotics use in the previous 30 days; or (4) they had hepatobiliary malignancy other than HCC.

This study was conducted in accordance with the ethical principles for medical research involving human subjects of the Declaration of Helsinki (updated version, 2013), and recommendations outlined in the International Conference on Harmonization guideline on Good Clinical Practice Guideline. Our study was approved by the Institutional Review Board of National Taiwan University Hospital (approval numbers: 201809039RIND and 201912040RINA). Written informed consent was obtained from all participants.

### Laboratory measurements

Baseline biochemical values including alanine aminotransferase (ALT), aspartate aminotransferase (AST), blood urea nitrogen (BUN), creatinine (CRE), fasting blood glucose (GLU-AC), platelet count, total cholesterol (T-CHO), high-density lipoprotein (HDL), low**-**density lipoprotein (LDL), and triglyceride (TG) were collected at enrollment. Laboratory assays were performed using commercially standardized automated techniques. The serum levels of HBeAg, anti-HBe, anti-HBs, anti-HCV, and anti-HDV were tested using commercial kits (Abbott Laboratories, Abbott Park, IL, USA).

### Fecal samples collection and DNA extraction

All fecal samples were frozen at − 20 °C immediately after collection by the participants at enrollment and were transported to the laboratory under frozen status. Bacterial DNA was extracted using the QIAamp PowerFecal Pro DNA Kit (Qiagen, Valencia, CA, USA), according to the manufacturer’s instructions. After extraction, aliquoted DNA samples were stored at – 80 ℃ until retrieval for this study. DNA concentration and integrity were evaluated using NanoDrop 2000 spectrophotometer (Thermo Fisher Scientific, Hudson, NH, United States) and through agarose gel electrophoresis, respectively.

### 16S rRNA gene sequencing

Two-step polymerase chain reaction was performed for library preparation according to the 16S library preparation guide published by Illumina. Primer pair sequences (forward: 5′- TCGTCGGCAGCGTCAGATGTGTATAAGAGACAGCCTACGGGNGGCWGCAG-3′; reverse: 5′- GTCTCGTGGGCTCGGAGATGTGTATAAGAGACAGGACTACHVGGGTATCTAATCC-3′) were used to sequence the V3 and V4 regions of the bacterial 16S rRNA gene. Dual indices and Illumina sequencing adapters were attached through polymerase chain reaction using Nextera XT DNA Library Preparation Kit according to the manufacturer’s protocol. Library quantification was performed for quality control before sequencing by Agilent Technologies 2100 Bioanalyzer. Then, the pooled libraries were sequenced on the Illumina MiSeq platform with v3 reagents (paired-end sequencing; 2 × 300 bps) [36].

### Bioinformatics and statistical analysis

The DADA2 and QIIME 2 (version 2019.10) pipelines were used for sequence denoising and further processing [37, 38]. The taxonomy was assigned using a naïve Bayes classifier trained on the SILVA 138 99% full-length 16S rRNA gene sequence database. Samples were rarefied to 46,986 reads through random sampling, and alpha and beta diversities were measured using the R (version 4.1.3; the R Project for Statistical Computing) package phyloseq [39].

Alpha diversity parameters, such as observed ASVs, Shannon diversity, Simpson diversity, Chao1 index, and Chao2 index, were assessed to estimate species evenness and richness. The R package ggvenn was used to create the corresponding Venn diagram. The Kruskal–Wallis test was performed to compare the study groups in terms of alpha diversity. Beta diversity matrices (weighted and unweighted UniFrac distances) were generated, and principal coordinate analysis (PCoA) was performed to estimate the between-group dissimilarities in microbiota composition. The permutational multivariate analysis of variance (PERMANOVA) with 999 permutations was performed using the Adonis2 function and pairwiseAdonis package in R was used to analyze the statistical significance of beta diversity. The results of alpha and beta diversity analyses were visualized using the R package ggplot2.

Linear discriminant analysis (LDA) effect size (LEfSe) was performed using the Galaxy browser (http://huttenhower.sph.harvard.edu/galaxy/) to identify the taxa that were most likely to be associated with between-group differences [40]. Phylogenetic Investigation of Communities by Reconstruction of Unobserved States 2 (PICRUSt2) was used to predict microbial community functions on the basis of the Kyoto Encyclopedia of Genes and Genomes (KEGG) database [41, 42]. Kruskal–Wallis test was used for the between-group comparisons of the differential expression of Level 2 KEGG pathways.

Continuous data are presented in terms of median (interquartile range) values, and categorical data are presented in terms of number (percentage). Between-group differences were assessed using the Kruskal–Wallis, analysis of variance, or chi-square tests, as appropriate. The Benjamini–Hochberg procedure was used to control the false discovery rate. The Jonckheere–Terpstra test was used to evaluate trends of microbial abundance across different disease statuses. Statistical analysis was performed using STATA (version 16.0; Stata Corporation, College Station, TX, USA) and R. All tests were two-sided and a *P* value < 0.05 indicated statistical significance.

## Supplementary Information

**Additional file 1: Table S1.** Statistical significance of beta diversity distance matrices.

**Additional file 2: Table S2.** Differentially abundant phyla between groups identified by LEfSe.

**Additional file 3: Table S3.** The average abundance of predicted Level 2 KEGG pathways in each group.

**Additional file 4: Table S4.** Comparison of Level 2 KEGG pathways.

**Additional file 5: Figure S1.** Comparisons of bacterial diversity and richness of patients with HBeAg (+) chronic HBV infection, HBeAg (+) chronic hepatitis B, HBeAg (−) chronic HBV infection, HBeAg (−) chronic hepatitis B, and resolved HBV. **A** A Venn diagram displays the unique and shared ASVs among the four groups. **B** The HBeAg (−) chronic hepatitis B group had the least observed ASVs among the five groups. The HBeAg (−) chronic hepatitis B group had the lowest alpha diversity indices, including **C** Shannon diversity, **D** Simpson diversity, **E** Chao1 index, and **F** Chao2 index. * means *P* < 0.05 and ** means *P* < 0.01.

**Additional file 6: Figure S2.** Beta diversity indices of patients with HBeAg (+) chronic HBV infection, HBeAg (+) chronic hepatitis B, HBeAg (−) chronic HBV infection, HBeAg (−) chronic hepatitis B, and resolved HBV. **A** PCoA plot of bacterial beta diversity based on the weighted UniFrac distance. **B** PCoA plot of bacterial beta diversity based on the unweighted UniFrac distance. No separate cluster was found between HBeAg (+) chronic HBV infection, HBeAg (+) chronic hepatitis B, HBeAg (−) chronic HBV infection, HBeAg (−) chronic hepatitis B, and resolved HBV.

**Additional file 7: Table S5.** Statistical significance of beta diversity distance matrices among patients with HBeAg (+) chronic HBV infection, HBeAg (+) chronic hepatitis B, HBeAg (−) chronic HBV infection, HBeAg (−) chronic hepatitis B, and resolved HBV.

## Data Availability

The datasets generated and/or analysed during the current study are available in the National Center for Biotechnology Information (NCBI) database repository, [https://www.ncbi.nlm.nih.gov/bioproject/PRJNA872871].

## Abbreviations

ALT: Alanine aminotransferase
AST: Aspartate aminotransferase
ASV: Amplicon sequence variant
BMI: Body mass index
BUN: Blood urea nitrogen
CHB: Chronic hepatitis B
CRE: Creatinine
F/B ratio: Firmicutes/Bacteroidetes ratio
GLU-AC: Fasting blood glucose
HBeAg: Hepatitis B e antigen
HBsAg: Hepatitis B surface antigen
HBV: Hepatitis B virus
HCC: Hepatocellular carcinoma
HCV: Hepatitis C virus
HDL: High-density lipoprotein
HDV: Hepatitis D virus
LC: Liver cirrhosis
LDL: Low-density lipoprotein
OTU: Operational taxonomic unit
SCFA: Short-chain fatty acid
T-CHO: Total cholesterol
TG: Triglyceride
