# Correction: Diversity and composition of gut microbiota in healthy individuals and patients at different stages of hepatitis B virus-related liver disease

**DOI:** 10.1186/s13099-023-00555-y

**Published:** 2023-06-17

**Authors:** Meng-Ju Lin, Tung-Hung Su, Chieh-Chang Chen, Wei-Kai Wu, Shih-Jer Hsu, Tai-Chung Tseng, Sih-Han Liao, Chun-Ming Hong, Hung-Chih Yang, Chun-Jen Liu, Ming-Shiang Wu, Jia-Horng Kao

**Affiliations:** 1 School of Medicine, College of Medicine, National Taiwan University, Taipei, Taiwan; 2 Division of Gastroenterology and Hepatology, Department of Internal Medicine, National Taiwan University Hospital, 1 Chang‑Te Street, Taipei, 10048 Taiwan; 3 Hepatitis Research Center, National Taiwan University Hospital, Taipei, Taiwan; 4 Department of Medical Research, National Taiwan University Hospital, Taipei, Taiwan; 5 Section of Gastroenterology, Department of Medicine, National Taiwan University Cancer Center, Taipei, Taiwan; 6 Division of Hospital Medicine, Department of Internal Medicine, National Taiwan University Hospital, Taipei, Taiwan; 7 Graduate Institute of Clinical Medicine, College of Medicine, National Taiwan University, 1 Chang‑Te Street, Taipei, 10048 Taiwan

**Correction: Gut Pathogens (2023) 15:24** **https://doi.org/10.1186/s13099-023-00549-w**

Following publication of the original article [1], it was noted that due to a typesetting mistake, errors were introduced in the caption of Additional files 5–7 in the Supplementary information.

The publisher apologises to the authors and readers for the inconvenience caused by the error.

That is,

- “HBeAgchronic HBV infection” should be “HBeAg (+) chronic HBV infection”
- “HBeAgchronic hepatitis B” should be “HBeAg (+) chronic hepatitis B”
- “HBeAgchronic HBV infection” should be “HBeAg (−) chronic HBV infection”
- “HBeAgchronic hepatitis B” should be “HBeAg (−) chronic hepatitis B”

It has been corrected in this correction. The original article has been updated.

**Additional file 5: Figure S1.**

Comparisons of bacterial diversity and richness of patients with HBeAg (+) chronic HBV infection, HBeAg (+) chronic hepatitis B, HBeAg (−) chronic HBV infection, HBeAg (−) chronic hepatitis B, and resolved HBV. **A** A Venn diagram displays the unique and shared ASVs among the four groups. **B** The HBeAg (−) chronic hepatitis B group had the least observed ASVs among the five groups. The HBeAg (−) chronic hepatitis B group had the lowest alpha diversity indices, including **C** Shannon diversity, **D** Simpson diversity, **E** Chao1 index, and **F** Chao2 index. * means P < 0.05 and ** means P < 0.01.

**Additional file 6: Figure S2.**

Beta diversity indices of patients with HBeAg (+) chronic HBV infection, HBeAg (+) chronic hepatitis B, HBeAg (−) chronic HBV infection, HBeAg (−) chronic hepatitis B, and resolved HBV. **A** PCoA plot of bacterial beta diversity based on the weighted UniFrac distance. **B** PCoA plot of bacterial beta diversity based on the unweighted UniFrac distance. No separate cluster was found between HBeAg (+) chronic HBV infection, HBeAg (+) chronic hepatitis B, HBeAg (−) chronic HBV infection, HBeAg (−) chronic hepatitis B, and resolved HBV.

**Additional file 7: Table S5.**

Statistical significance of beta diversity distance matrices among patients with HBeAg (+) chronic HBV infection, HBeAg (+) chronic hepatitis B, HBeAg (−) chronic HBV infection, HBeAg (−) chronic hepatitis B, and resolved HBV.
