# Supplementary material for: Diversity and composition of gut microbiota in healthy individuals and patients at different stages of hepatitis B virus-related liver disease
Source: Gut Pathog. 2023 May 22;15:24. doi: 10.1186/s13099-023-00549-w (PMC10201741; doi:10.1186/s13099-023-00549-w)
Supplement: Supplementary file 1 — Additional file 1: Table S1. Statistical significance of beta diversity distance matrices. [file 13099_2023_549_MOESM1_ESM.docx]

| Beta diversity indices | F. Model | R2 | *P* value | q value |
| --- | --- | --- | --- | --- |
| **Weighted Unifrac distance** |  |  |  |  |
| Resolved HBV vs. Healthy control | 1.610 | 0.023 | 0.019 | 0.038 |
| Chronic hepatitis B vs. Healthy control | 2.402 | 0.021 | 0.001 | 0.003 |
| Advanced liver disease vs. Healthy control | 3.221 | 0.035 | 0.001 | 0.003 |
| Chronic hepatitis B vs. Resolved HBV | 0.973 | 0.014 | 0.501 | 0.501 |
| Advanced liver disease vs. Resolved HBV | 1.261 | 0.027 | 0.116 | 0.174 |
| Advanced liver disease vs. Chronic hepatitis B | 1.051 | 0.012 | 0.343 | 0.412 |
| **Unweighted Unifrac distance** |  |  |  |  |
| Resolved HBV vs. Healthy control | 1.827 | 0.026 | 0.001 | 0.002 |
| Chronic hepatitis B vs. Healthy control | 2.777 | 0.024 | 0.001 | 0.002 |
| Advanced liver disease vs. Healthy control | 3.091 | 0.034 | 0.001 | 0.002 |
| Chronic hepatitis B vs. Resolved HBV | 0.790 | 0.011 | 0.996 | 0.996 |
| Advanced liver disease vs. Resolved HBV | 0.875 | 0.019 | 0.901 | 0.996 |
| Advanced liver disease vs. Chronic hepatitis B | 1.159 | 0.013 | 0.082 | 0.123 |

Table S1. Statistical significance of beta diversity distance matrices
