# Supplementary material for: Diversity and composition of gut microbiota in healthy individuals and patients at different stages of hepatitis B virus-related liver disease
Source: Gut Pathog. 2023 May 22;15:24. doi: 10.1186/s13099-023-00549-w (PMC10201741; doi:10.1186/s13099-023-00549-w)
Supplement: Supplementary file 2 — Additional file 2: Table S2. Differentially abundant phyla between groups identified by LEfSe. [file 13099_2023_549_MOESM2_ESM.docx]

| Phylum | Enriched group | LDA score (log10) | *P* |
| --- | --- | --- | --- |
| Resolved HBV vs. Healthy control | | | |
| Bacteroidetes | Healthy control | 5.04 | <0.001 |
| Firmicutes | Resolved HBV | 4.96 | <0.001 |
| Verrucomicrobiota | Resolved HBV | 4.02 | 0.004 |
| Fusobacteria | Resolved HBV | 3.55 | 0.007 |
| Chronic hepatitis B vs. Healthy control | | | |
| Bacteroidetes | Healthy control | 4.96 | <0.001 |
| Firmicutes | Chronic hepatitis B | 4.77 | <0.001 |
| Fusobacteria | Chronic hepatitis B | 3.87 | 0.004 |
| Verrucomicrobiota | Chronic hepatitis B | 3.67 | 0.006 |
| Advanced liver disease vs. Healthy control | | | |
| Bacteroidetes | Healthy control | 5.07 | <0.001 |
| Firmicutes | Advanced liver disease | 4.82 | <0.001 |
| Proteobacteria | Advanced liver disease | 4.38 | 0.007 |
| Actinobacteriota | Advanced liver disease | 4.16 | 0.047 |
| Verrucomicrobiota | Advanced liver disease | 3.89 | 0.006 |
| Fusobacteria | Advanced liver disease | 3.65 | <0.001 |
| Advanced liver disease vs. Chronic hepatitis B | | | |
| Proteobacteria | Advanced liver disease | 4.06 | 0.043 |

Table S2. Differentially abundant phyla between groups identified by LEfSe (only logarithmic LDA scores > 3.0 are shown)
