# Supplementary material for: Diversity and composition of gut microbiota in healthy individuals and patients at different stages of hepatitis B virus-related liver disease
Source: Gut Pathog. 2023 May 22;15:24. doi: 10.1186/s13099-023-00549-w (PMC10201741; doi:10.1186/s13099-023-00549-w)
Supplement: Supplementary file 3 — Additional file 3: Table S3. The average abundance of predicted Level 2 KEGG pathways in each group. [file 13099_2023_549_MOESM3_ESM.docx]

Table S3. The average abundance of predicted Level 2 KEGG pathways in each group

| Level 2 KEGG pathways | Healthy control | Resolved HBV | Chronic hepatitis B | Advanced liver disease |
| --- | --- | --- | --- | --- |
| Aging | 0.0044 | 0.0044 | 0.0044 | 0.0045 |
| Amino acid metabolism | 0.1142 | 0.1151 | 0.1132 | 0.1130 |
| Biosynthesis of other secondary metabolites | 0.0295 | 0.0266 | 0.0282 | 0.0276 |
| Cancer: overview | 0.0094 | 0.0100 | 0.0096 | 0.0097 |
| Cancer: specific types | 0.0008 | 0.0009 | 0.0009 | 0.0009 |
| Carbohydrate metabolism | 0.1787 | 0.1727 | 0.1757 | 0.1760 |
| Cardiovascular disease | 0.0062 | 0.0063 | 0.0063 | 0.0062 |
| Cell growth and death | 0.0140 | 0.0122 | 0.0131 | 0.0128 |
| Cell motility | 0.0073 | 0.0072 | 0.0092 | 0.0090 |
| Cellular community - eukaryotes | 0.0000 | 0.0000 | 0.0000 | 0.0000 |
| Cellular community - prokaryotes | 0.0299 | 0.0329 | 0.0331 | 0.0337 |
| Circulatory system | 0.0000 | 0.0000 | 0.0000 | 0.0000 |
| Development and regeneration | 0.0004 | 0.0003 | 0.0003 | 0.0003 |
| Digestive system | 0.0018 | 0.0020 | 0.0019 | 0.0019 |
| Drug resistance: antimicrobial | 0.0189 | 0.0188 | 0.0184 | 0.0185 |
| Drug resistance: antineoplastic | 0.0031 | 0.0033 | 0.0031 | 0.0032 |
| Endocrine and metabolic disease | 0.0035 | 0.0035 | 0.0037 | 0.0037 |
| Endocrine system | 0.0126 | 0.0111 | 0.0121 | 0.0118 |
| Energy metabolism | 0.0708 | 0.0688 | 0.0696 | 0.0691 |
| Environmental adaptation | 0.0043 | 0.0037 | 0.0038 | 0.0037 |
| Excretory system | 0.0003 | 0.0005 | 0.0003 | 0.0003 |
| Folding, sorting and degradation | 0.0241 | 0.0241 | 0.0238 | 0.0237 |
| Glycan biosynthesis and metabolism | 0.0506 | 0.0470 | 0.0450 | 0.0447 |
| Immune disease | 0.0007 | 0.0006 | 0.0007 | 0.0007 |
| Immune system | 0.0042 | 0.0037 | 0.0040 | 0.0039 |
| Infectious disease: bacterial | 0.0107 | 0.0111 | 0.0111 | 0.0112 |
| Infectious disease: parasitic | 0.0004 | 0.0006 | 0.0005 | 0.0006 |
| Infectious disease: viral | 0.0007 | 0.0007 | 0.0007 | 0.0008 |
| Information processing in viruses | 0.0001 | 0.0002 | 0.0002 | 0.0002 |
| Lipid metabolism | 0.0327 | 0.0305 | 0.0315 | 0.0313 |
| Membrane transport | 0.0466 | 0.0561 | 0.0551 | 0.0569 |
| Metabolism of cofactors and vitamins | 0.0723 | 0.0752 | 0.0704 | 0.0707 |
| Metabolism of other amino acids | 0.0232 | 0.0236 | 0.0234 | 0.0236 |
| Metabolism of terpenoids and polyketides | 0.0145 | 0.0147 | 0.0149 | 0.0147 |
| Nervous system | 0.0039 | 0.0035 | 0.0036 | 0.0036 |
| Neurodegenerative disease | 0.0040 | 0.0044 | 0.0040 | 0.0041 |
| Nucleotide metabolism | 0.0460 | 0.0465 | 0.0465 | 0.0463 |
| Replication and repair | 0.0483 | 0.0478 | 0.0484 | 0.0479 |
| Signal transduction | 0.0327 | 0.0349 | 0.0346 | 0.0353 |
| Signaling molecules and interaction | 0.0000 | 0.0000 | 0.0000 | 0.0000 |
| Substance dependence | 0.0000 | 0.0000 | 0.0000 | 0.0000 |
| Transcription | 0.0023 | 0.0024 | 0.0025 | 0.0024 |
| Translation | 0.0518 | 0.0513 | 0.0527 | 0.0515 |
| Transport and catabolism | 0.0083 | 0.0065 | 0.0064 | 0.0064 |
| Xenobiotics biodegradation and metabolism | 0.0117 | 0.0147 | 0.0132 | 0.0138 |
