# Supplementary material for: Diversity and composition of gut microbiota in healthy individuals and patients at different stages of hepatitis B virus-related liver disease
Source: Gut Pathog. 2023 May 22;15:24. doi: 10.1186/s13099-023-00549-w (PMC10201741; doi:10.1186/s13099-023-00549-w)
Supplement: Supplementary file 4 — Additional file 4: Table S4. Comparison of Level 2 KEGG pathways. [file 13099_2023_549_MOESM4_ESM.docx]

Table S4. Comparison of Level 2 KEGG pathways

| Level 2 KEGG pathways | *P* value | Resolved HBV vs. Healthy control | Chronic hepatitis B vs. Healthy control | Advanced liver disease vs. Healthy control | Chronic hepatitis B vs. Resolved HBV | Advanced liver disease vs. Resolved HBV | Advanced liver disease vs. Chronic hepatitis B |
| --- | --- | --- | --- | --- | --- | --- | --- |
| Aging | 0.4545 | 0.527 | 0.910 | 0.974 | 0.780 | 0.398 | 0.745 |
| Amino acid metabolism | 0.0717 | 0.537 | 0.371 | 0.382 | 0.088 | 0.090 | 0.997 |
| Biosynthesis of other secondary metabolites | 0.0004 | 0.231 | 0.010 | 0.000 | 1.000 | 0.749 | 0.525 |
| Cancer: overview | 0.0717 | 0.699 | 0.243 | 0.020 | 1.000 | 0.757 | 0.548 |
| Cancer: specific types | 0.0011 | 0.685 | 0.045 | 0.001 | 0.947 | 0.413 | 0.430 |
| Carbohydrate metabolism | 0.0066 | 0.079 | 0.035 | 0.146 | 0.897 | 0.852 | 0.996 |
| Cardiovascular disease | 0.7532 | 0.771 | 0.896 | 1.000 | 0.953 | 0.774 | 0.903 |
| Cell growth and death | 0.0004 | 0.045 | 0.000 | 0.000 | 0.988 | 0.670 | 0.622 |
| Cell motility | 0.0085 | 0.144 | 0.009 | 0.066 | 0.999 | 0.989 | 0.993 |
| Cellular community - eukaryotes | 0.5772 | 0.871 | 0.497 | 0.683 | 0.999 | 1.000 | 1.000 |
| Cellular community - prokaryotes | 0.0004 | 0.092 | 0.000 | 0.000 | 0.935 | 0.740 | 0.904 |
| Circulatory system | 0.0048 | 0.549 | 0.030 | 0.098 | 0.974 | 0.986 | 1.000 |
| Development and regeneration | 0.0310 | 0.111 | 0.019 | 0.419 | 0.976 | 0.703 | 0.742 |
| Digestive system | 0.2925 | 1.000 | 0.447 | 0.307 | 0.809 | 0.659 | 0.968 |
| Drug resistance: antimicrobial | 0.0392 | 0.021 | 0.053 | 0.490 | 0.562 | 0.292 | 0.852 |
| Drug resistance: antineoplastic | 0.5554 | 0.974 | 0.977 | 0.962 | 0.998 | 0.882 | 0.832 |
| Endocrine and metabolic disease | 0.0180 | 0.137 | 0.012 | 0.063 | 0.996 | 0.988 | 0.998 |
| Endocrine system | 0.0004 | 0.388 | 0.005 | 0.000 | 0.953 | 0.419 | 0.420 |
| Energy metabolism | 0.0017 | 0.215 | 0.045 | 0.006 | 0.991 | 0.967 | 0.704 |
| Environmental adaptation | 0.0004 | 0.001 | 0.000 | 0.000 | 0.980 | 0.993 | 0.788 |
| Excretory system | 0.9024 | 0.999 | 0.985 | 0.940 | 1.000 | 0.954 | 0.815 |
| Folding, sorting and degradation | 0.5947 | 0.913 | 0.471 | 0.300 | 0.399 | 0.269 | 0.957 |
| Glycan biosynthesis and metabolism | 0.0004 | 0.004 | 0.000 | 0.000 | 0.999 | 1.000 | 0.994 |
| Immune disease | 0.0137 | 0.076 | 0.871 | 0.095 | 0.209 | 0.901 | 0.336 |
| Immune system | 0.1063 | 0.921 | 0.438 | 0.058 | 0.393 | 0.095 | 0.598 |
| Infectious disease: bacterial | 0.0225 | 0.935 | 0.027 | 0.019 | 0.637 | 0.461 | 0.954 |
| Infectious disease: parasitic | 0.0004 | 0.138 | 0.000 | 0.000 | 0.891 | 0.718 | 0.943 |
| Infectious disease: viral | 0.0227 | 0.153 | 0.136 | 0.023 | 0.874 | 1.000 | 0.737 |
| Information processing in viruses | 0.3003 | 0.793 | 0.477 | 0.380 | 1.000 | 0.996 | 0.983 |
| Lipid metabolism | 0.0004 | 0.005 | 0.000 | 0.000 | 0.923 | 0.998 | 0.937 |
| Membrane transport | 0.0004 | 0.033 | 0.000 | 0.000 | 0.972 | 0.725 | 0.787 |
| Metabolism of cofactors and vitamins | 0.0088 | 1.000 | 0.021 | 0.173 | 0.309 | 0.553 | 0.969 |
| Metabolism of other amino acids | 0.4786 | 0.766 | 0.921 | 0.517 | 0.518 | 0.252 | 0.838 |
| Metabolism of terpenoids and polyketides | 0.0101 | 0.048 | 0.003 | 0.531 | 0.980 | 0.419 | 0.317 |
| Nervous system | 0.0077 | 0.321 | 0.023 | 0.007 | 1.000 | 0.918 | 0.848 |
| Neurodegenerative disease | 0.9024 | 0.944 | 0.994 | 0.815 | 0.978 | 1.000 | 0.908 |
| Nucleotide metabolism | 0.4545 | 0.725 | 0.301 | 0.834 | 1.000 | 0.978 | 0.908 |
| Replication and repair | 0.5947 | 0.733 | 0.992 | 0.934 | 0.830 | 0.518 | 0.832 |
| Signal transduction | 0.0077 | 0.844 | 0.069 | 0.022 | 0.886 | 0.621 | 0.865 |
| Signaling molecules and interaction | 0.9819 | 0.863 | 0.533 | 0.956 | 1.000 | 0.981 | 0.913 |
| Substance dependence | 0.0174 | 0.770 | 0.665 | 0.393 | 0.334 | 0.998 | 0.052 |
| Transcription | 0.0353 | 0.098 | 0.031 | 0.365 | 0.936 | 0.710 | 0.871 |
| Translation | 0.3886 | 0.569 | 0.743 | 0.993 | 0.912 | 0.498 | 0.655 |
| Transport and catabolism | 0.0004 | 0.003 | 0.000 | 0.000 | 0.985 | 0.990 | 1.000 |
| Xenobiotics biodegradation and metabolism | 0.0004 | 0.598 | 0.001 | 0.000 | 0.684 | 0.190 | 0.488 |

All *P* values reported were adjusted by the Benjamini-Hochberg procedure.
