# Supplementary figures and images for: Diversity and composition of gut microbiota in healthy individuals and patients at different stages of hepatitis B virus-related liver disease
Source: Gut Pathog. 2023 May 22;15:24. doi: 10.1186/s13099-023-00549-w (PMC10201741; doi:10.1186/s13099-023-00549-w)

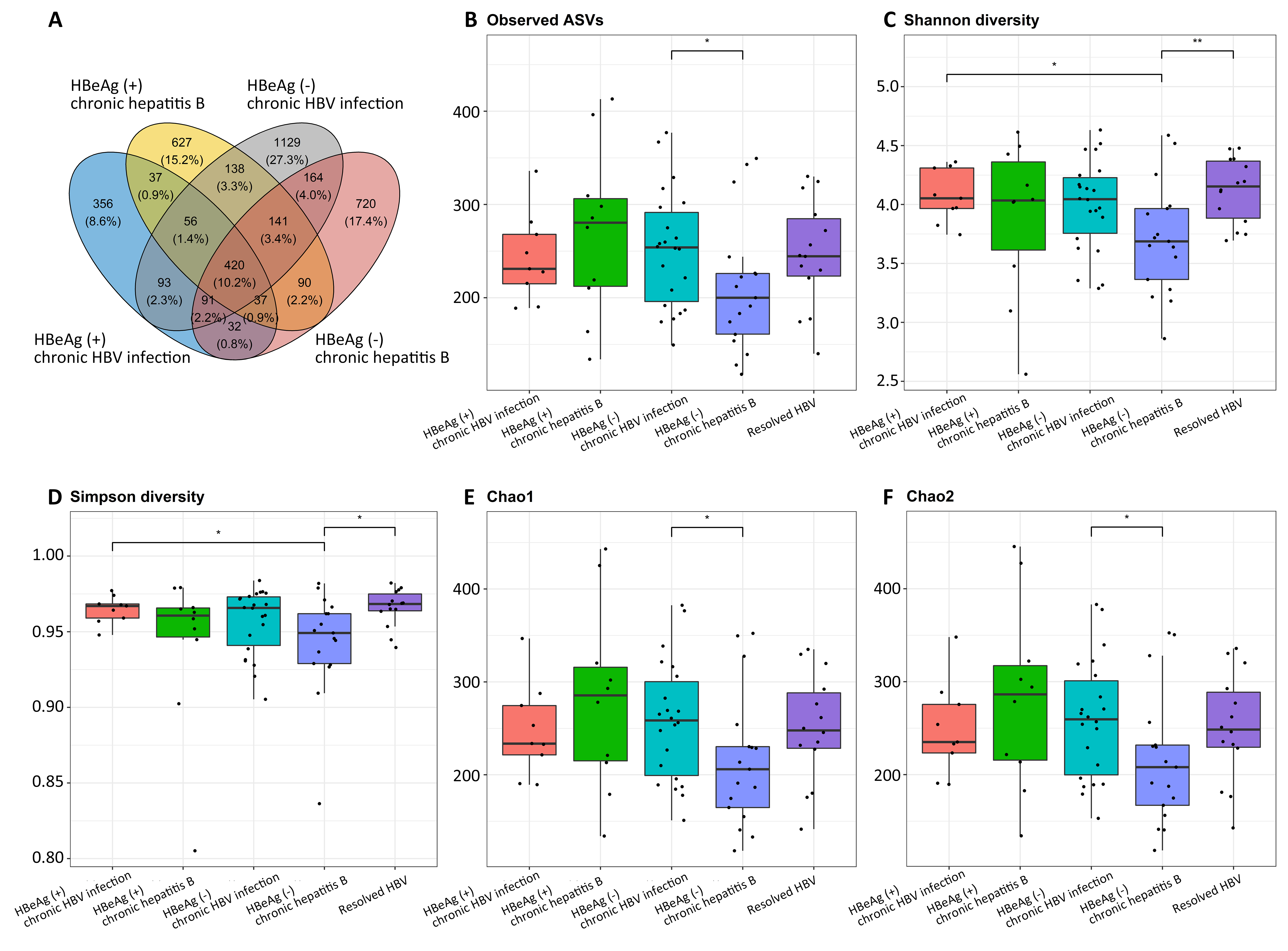

Supplement: Supplementary file 5 — Additional file 5: Figure S1. Comparisons of bacterial diversity and richness of patients with HBeAg (+) chronic HBV infection, HBeAg (+) chronic hepatitis B, HBeAg (−) chronic HBV infection, HBeAg (−) chronic hepatitis B, and resolved HBV. A A Venn diagram displays the unique and shared ASVs among the four groups. B The HBeAg (−) chronic hepatitis B group had the least observed ASVs among the five groups. The HBeAg (−) chronic hepatitis B group had the lowest alpha diversity indices, including C Shannon diversity, D Simpson diversity, E Chao1 index, and F Chao2 index. * means P < 0.05 and ** means P < 0.01. [file 13099_2023_549_MOESM5_ESM.tif]

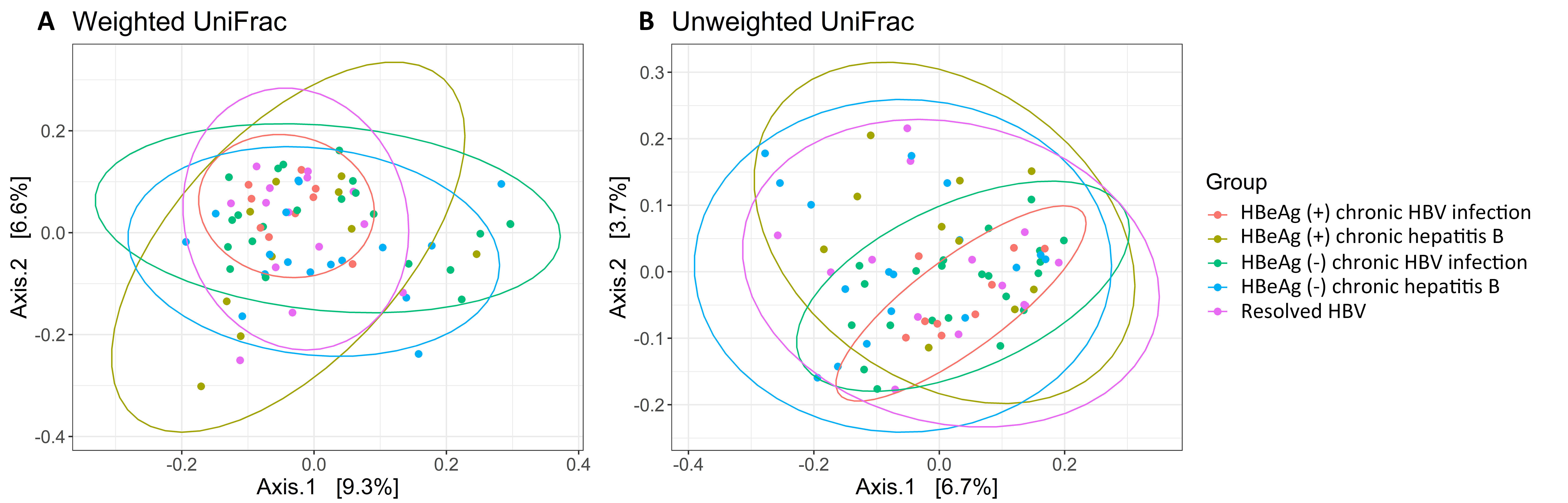

Supplement: Supplementary file 6 — Additional file 6: Figure S2. Beta diversity indices of patients with HBeAg (+) chronic HBV infection, HBeAg (+) chronic hepatitis B, HBeAg (−) chronic HBV infection, HBeAg (−) chronic hepatitis B, and resolved HBV. A PCoA plot of bacterial beta diversity based on the weighted UniFrac distance. B PCoA plot of bacterial beta diversity based on the unweighted UniFrac distance. No separate cluster was found between HBeAg (+) chronic HBV infection, HBeAg (+) chronic hepatitis B, HBeAg (−) chronic HBV infection, HBeAg (−) chronic hepatitis B, and resolved HBV. [file 13099_2023_549_MOESM6_ESM.tif]
