# Supplementary material for: Diversity and composition of gut microbiota in healthy individuals and patients at different stages of hepatitis B virus-related liver disease
Source: Gut Pathog. 2023 May 22;15:24. doi: 10.1186/s13099-023-00549-w (PMC10201741; doi:10.1186/s13099-023-00549-w)
Supplement: Supplementary file 7 — Additional file 7: Table S5. Statistical significance of beta diversity distance matrices among patients with HBeAg (+) chronic HBV infection, HBeAg (+) chronic hepatitis B, HBeAg (−) chronic HBV infection, HBeAg (−) chronic hepatitis B, and resolved HBV. [file 13099_2023_549_MOESM7_ESM.docx]

Table S5. Statistical significance of beta diversity distance matrices among patients with HBeAg (+) chronic HBV infection, HBeAg (+) chronic hepatitis B, HBeAg (−) chronic HBV infection, HBeAg (−) chronic hepatitis B, and resolved HBV

| Beta diversity indices | F. Model | R2 | *P* value | q value |
| --- | --- | --- | --- | --- |
| **Weighted Unifrac distance** |  |  |  |  |
| HBeAg (+) chronic HBV infection vs. HBeAg (+) chronic hepatitis B | 1.165 | 0.064 | 0.152 | 0.566 |
| HBeAg (+) chronic HBV infection vs. HBeAg (−) chronic HBV infection | 1.007 | 0.034 | 0.453 | 0.566 |
| HBeAg (+) chronic HBV infection vs. HBeAg (−) chronic hepatitis B | 1.195 | 0.047 | 0.176 | 0.566 |
| HBeAg (+) chronic HBV infection vs. Resolved HBV | 1.354 | 0.061 | 0.057 | 0.566 |
| HBeAg (+) chronic hepatitis B vs. HBeAg (−) chronic HBV infection | 0.919 | 0.030 | 0.644 | 0.644 |
| HBeAg (+) chronic hepatitis B vs. HBeAg (−) chronic hepatitis B | 0.968 | 0.037 | 0.530 | 0.589 |
| HBeAg (+) chronic hepatitis B vs. Resolved HBV | 1.034 | 0.045 | 0.402 | 0.566 |
| HBeAg (−) chronic HBV infection vs. HBeAg (−) chronic hepatitis B | 1.054 | 0.028 | 0.364 | 0.566 |
| HBeAg (−) chronic HBV infection vs. Resolved HBV | 1.009 | 0.029 | 0.442 | 0.566 |
| HBeAg (−) chronic hepatitis B vs. Resolved HBV | 1.057 | 0.035 | 0.345 | 0.566 |
| **Unweighted Unifrac distance** |  |  |  |  |
| HBeAg (+) chronic HBV infection vs. HBeAg (+) chronic hepatitis B | 1.156 | 0.064 | 0.079 | 0.243 |
| HBeAg (+) chronic HBV infection vs. HBeAg (−) chronic HBV infection | 1.113 | 0.037 | 0.097 | 0.243 |
| HBeAg (+) chronic HBV infection vs. HBeAg (−) chronic hepatitis B | 1.207 | 0.048 | 0.065 | 0.243 |
| HBeAg (+) chronic HBV infection vs. Resolved HBV | 1.005 | 0.046 | 0.439 | 0.627 |
| HBeAg (+) chronic hepatitis B vs. HBeAg (−) chronic HBV infection | 1.089 | 0.035 | 0.159 | 0.318 |
| HBeAg (+) chronic hepatitis B vs. HBeAg (−) chronic hepatitis B | 1.059 | 0.041 | 0.266 | 0.443 |
| HBeAg (+) chronic hepatitis B vs. Resolved HBV | 0.975 | 0.042 | 0.566 | 0.629 |
| HBeAg (−) chronic HBV infection vs. HBeAg (−) chronic hepatitis B | 1.120 | 0.031 | 0.062 | 0.243 |
| HBeAg (−) chronic HBV infection vs. Resolved HBV | 0.881 | 0.025 | 0.919 | 0.919 |
| HBeAg (−) chronic hepatitis B vs. Resolved HBV | 0.976 | 0.033 | 0.503 | 0.629 |
